# Socioeconomic inequalities in cigarette smoking among men: evidence from the 2003 and 2008 Ghana demographic and health surveys

**DOI:** 10.1186/0778-7367-71-9

**Published:** 2013-04-26

**Authors:** David Doku, Eugene Kofuor Maafo Darteh, Akwasi Kumi-Kyereme

**Affiliations:** 1 Department of Population and Health, University of Cape Coast, Private Mail Bag, University Post Office, Cape Coast, Ghana

**Keywords:** Cigarette smoking, Smoking epidemic, Diffusion of innovations, Ghanaian men

## Abstract

**Background:**

Tobacco use is a public health burden in both developed and developing countries. However, there is still a dearth of nationally representative studies from Sub-Saharan Africa to inform interventions in the region. Socioeconomic trends and disparities in cigarette smoking were explored among Ghanaian men.

**Method:**

A nationally representative sample of Ghanaian men 15–59 years was surveyed in the 2003 (N = 5015) and 2008 (N = 4568) Ghana Demographic and Health Surveys (N = 9583). Logistic regression analyses were conducted to investigate cigarette smoking by socioeconomic status (SES) and the changes over the two study periods. The results are presented as adjusted odds ratios (AOR) at 95% confidence intervals (CI)

**Results:**

The prevalence decreased by 1.7% from 9% (95% CI 0.09–0.11) in 2003 to 7.3% (95% CI 0.07–0.09) in 2008. The prevalence of cigarette smoking was higher in the older age groups (25–34 year-olds and 35–59 year-olds) compared to 15–24 year-olds. Education (AOR = 2.2, 95% CI 1.4–3.4; no education vs higher education) and occupation (AOR = 4.2, 95% CI 2.3–7.6; not working vs managerial position) and being in labour force (AOR = 2.6, 95% CI 1.7–4.0) were related to cigarette smoking. Furthermore, religion, wealth (AOR = 3.1 95% CI 2.1–4.5; poorest compared to richest) and rural residence (AOR = 1.8, 95% CI 1.5–2.1) were associated with cigarette smoking. Over the period, cigarette smoking seems to have decreased among Ghanaian male at the population level but not among all groups by age, education, wealth and place of residence.

**Conclusion:**

Cigarette smoking interventions should be structured to reduce the menace among men. Such interventions must also particularly target lower socioeconomic groups in order to avert an increase in the inequalities in the behaviour and prervent a consequent increase in the socioeconomic gradient in tobacco-related diseases and deaths.

## Background

Tobacco use has been a global public health menace for many decades and it accounts for the largest burden of preventable morbidity and mortality [1-3]. Over the past few decades, tobacco use has declined significantly and still continues to decline in most Western countries [4]. In most developing countries however, tobacco use has been rising in recent decades [5-7]. Current estimates are expected to increase, especially in developing countries as tobacco companies invade such places with aggressive marketing strategies to make up for the loss of markets in the developed countries.

The literature on tobacco use in Ghana is scanty. As it is in many African countries, studies conducted on tobacco use in Ghana mostly used small sample size and such studies were mostly conducted in the urban areas. An overview of the scant literature available suggests that the prevalence of smoking in Ghana is low [8,9]. In a survey conducted in one region (Ashanti region) out of the ten regions [10], it was found that 4% of Ghanaian adults smoke and that the smokers were mainly male dominant (9% males and 0.3% females). Efforts to ensure that the smoking prevalence is kept low will guarantee the prevention of scores of tobacco related morbidity and mortality and promote public health. Routine monitoring through surveillance, research and advocacy is one of such efforts.

Despite the low tobacco use in Ghana, the scant literature available suggests that the prevalence was higher in those with lower educational attainment compared with those with higher education [10]. In both developed and developing countries, apart from a few exceptions, most epidemiological studies have found consistent socioeconomic gradient in tobacco use [10-13]. Theoretically, it is argued that the spread of the tobacco epidemic is synonymous with the diffusion of innovation in society [14]. The epidemic first begins among high socioeconomic groups, diffuses to lower socioeconomic groups and recedes among high socioeconomic groups [14-16]. This theory has been used extensively to explain differences in smoking between the lower and upper socioeconomic groups in developed countries [15,16] but smoking in developing countries has not been examined in this perspective. Ezzati and Lopez [2] put forth that the smoking epidemic is in its early stages in many of the countries in Sub-Saharan Africa. Based on the postulation that the smoking epidemic is in its initial stages in developing countries, we expect increasing trends in cigarette smoking with no socioeconomic gradients because at the initial stage, cigarette smoking is scarce and no significant difference exists in socioeconomic groups.

The aim of this study is to explore socioeconomic disparities in cigarette smoking among Ghanaian men over a five year period.

## Methods

### Data collection

Data for this study came from the male questionnaire of the 2003 and 2008 Ghana Demographic and Health Surveys (GDHS). The Ghana Demographic Health Survey is a nationwide survey with a representative sample of women and men aged 15–49 and 15–59, respectively. A representative probability sample of about 6,600 households and 12,000 households were selected nationwide for the survey in 2003 and 2008, respectively. Both surveys used a two stage sample based on the 2000 Population and Housing Census to produce separate estimates for key indicators for each of the ten regions in Ghana. The first stage involved selecting sample points or clusters from an updated master sampling frame constructed from the 2000 Ghana Population and Housing Census. In both years, a total of 412 clusters were selected from the master sampling frame. The second stage of selection involved systematic sampling of 30 of the households listed in each cluster. This was done to ensure adequate numbers of completed individual interviews to provide estimates for key indicators with acceptable precision and to provide a sample large enough to identify adequate numbers of under-five deaths to provide data on causes of death. The clusters were selected using systematic sampling with probability proportional to size. Each household selected for the GDHS was eligible for interview with the household questionnaire. In half of the households selected for the survey, all eligible men aged 15–59-year-old were interviewed with the men’s questionnaire. In 2008, data was not administered in one cluster due to security concerns, thus final sample comprised of 12,323 selected households. The data collection took place over a three-month period, from early September to late November. Although the GDHS has been on-going every five years since 1988, questions on tobacco use were asked only in the 2003 (N = 5015) and the 2008 (N = 4568) surveys hence the present analysis is limited to the 2003 and 2008 questionnaire (N = 9583). The response rates were 93.8% and 95.8% for 2003 and 2008 respectively. The main reason for non-response was the failure to find individuals at home despite repeated visits to their household. Ethical approval for the study protocol was given by the Ghana Health Service Ethical Review Committee in Accra, Ghana.

The dependent variable used for the analyses was current cigarette smoking. It was assessed by the question, “Do you currently smoke cigarette or tobacco?” coded as for 0 “No” and 1 for “Yes”. For simplicity, in this study, we refer to current cigarette or tobacco use as *cigarette smoking*. The independent variables used in this study included occupation categorised as (not working, agriculture, manual, clerical or services, and professional and managerial), urban–rural residence and labour market position (categorised as “in labour force” and “not in labour force”). Labour market position refers to whether a respondent was in labour force or not. Those “in labour force” were those who reported that they were working and those “not in labour force” were those who reported that they were not working. The rest were household wealth, represented by wealth index (in five categories from poorest to richest). The wealth index was constructed using data on a household’s ownership of selected assets, such as televisions and bicycles; materials used for housing construction and types of water access and sanitation facilities. The wealth index was generated from principal components analysis. The index places individual households on a continuous scale of relative wealth. It was then categorized into five (poorest, poorer, middle, richer, and richest). In addition, education (coded as; no education, primary, secondary and higher) was used. The Ghanaian educational system has gone through a lot of changes over the years. However, generally, primary education is 6 years of schooling (from age 6 to age 12), secondary education is 6 years of schooling (from age 13 to age 18) and higher education from age 19 upwards.

### Statistical analysis

Binary logistic regression analyses were conducted to investigate the relationship between cigarette smoking and socioeconomic indicators, adjusted for age and study year. Next, multivariate analyses were conducted to investigate the independence of the association between cigarette smoking and the variables. Further, the interaction between age and the SES indicators were explored to ascertain the changes in the relationship between cigarette use over the five year period. Where statistically significant interactions between the SES indicators and the year were found, the relationships were further investigated graphically. Adjusted odds ratios (AOR) at 95% confidence intervals (CI) were reported for the logistic regression analyses. All analyses were weighted to account for sampling error due to the nested nature of individuals in households. SPSS version 20 was used in the analyses.

## Results

Among 15–59 year old participants, 5015 and 4568 men were interviewed in 2003 and 2008 respectively. The prevalence of cigarette smoking decreased by 1.7% from 9% (95% CI 0.09–0.11) in 2003 to 7.3% (95% CI 0.07–0.09) in 2008. The distribution of cigarette smoking by background characteristics are presented in Table 1. In the bivariate analysis, adjusted for age, several statistically significant socioeconomic differences in smoking were found (Table 1).

**Table 1:** The distribution of cigarette smoking by socio-demographic characteristics and bivariate odds ratios (OR) for cigarette smoking among Ghanaian men in the 2003 and 2008 demographic and health surveys

**Indicator**	**Cigarette smoking**		
	**2003**	**2008**	OR* (95% CI)
	**N (%)**	**N (%)**	**Total sample**
**Age (in years)**			
15–24	1791(1.6)	1613 (1.5)	1.0
25–34	1387 (10.1)	1156 (7.5)	6.1 (4.5–8.4)
35–59	1836 (19.5)	1796 (14.5)	11.8 (8.8–15.9)
**Education**			
Higher education	316 (6.0)	412 (4.6)	1.0
Secondary education	3014 (6.3)	2850 (4.9)	1.4 (1.0–1.9)
Primary education	803 (10.7)	663 (10.1)	2.9 (2.0–4.2)
No education	881 (26.2)	640 (23.0)	4.7 (3.3–6.6)
**Occupation**			
Managerial/professional	411 (8.2)	458 (10.3)	1.0
Clerical and services	515 (10.3)	784 (17.7)	1.7 (1.2–2.4)
Manual	975 (19.4)	805 (18.2)	1.6 (1.1–2.3)
Agriculture	1970 (39.9)	1567 (35.4)	3.0 (2.2–4.1)
No working	1061 (21.2)	809 (18.3)	0.6 (0.3–1.0)
**Religion**			
Christianity	4447 (8.3)	3304 (4.7)	1.0
Traditional religion	329 (25.5)	253 (25.7)	3.6 (2.9–4.5)
Muslim	238 (31.5)	755 (13.4)	2.9 (2.0–4.1)
Other religions	2 (0)	252 (19.8)	2.0 (1.6–2.4)
**Labour market position**			
Not in labour force	3790 (2.8)	3640 (2.5)	1.0
In labour force	1222 (13.0)	909 (9.6)	2.0 (1.5–2.7)
**Wealth index**			
Richest	1204 (5.7)	1079(3.3)	1.0
Richer	1060 (6.6)	1078 (5.2)	1.5 (1.1–1.9)
Middle	976 (8.5)	784 (7.9)	2.0 (1.1–2.9)
Poorer	903 (13.0)	815 (7.7)	2.4 (1.9–3.1)
Poorest	872 (21.6)	809 (19.2)	4.1 (3.2–5.3)
**Place of residence**			
Urban	2250 (7.0)	2122 (5.6)	1.0
Rural	2764 (13.3)	2443 (10.4)	1.8 (1.5–2.1)
**Survey year**			
2003			1.0
2008			0.7 (0.6–0.8)

*Age and year adjusted odds ratio.

Men aged 25–34 years and 35–59 years were more likely to smoke cigarettes compared to younger men (15–24 years old). Men without formal education (AOR =2.2, 95% CI 1.4–3.4) and those with secondary school education (AOR = 1.6, 95% CI 1.1–2.5) had higher likelihood of cigarette smoking compared to those with higher education (Table 2). Similarly, differences in cigarette smoking were found by occupational categories. Compared to those with professional and managerial occupations, men in clerical and services, agricultural and manual occupations were more likely to use cigarettes. Also, the likelihood of cigarette smoking varied by religious affiliations in such a way that Traditional Religion practitioners, Muslims and other religious groups were more likely to smoke cigarettes than their counterparts in the Christian faith. By labour market position, men who were in labour force had two-folds the possibility of cigarette smoking compared to those not in labour force. Furthermore, socioeconomic gradient was found in cigarette smoking by wealth. The poorest (AOR = 3.1, 95% CI 2.1–4.5), the poorer (AOR = 1.9, 95% CI 1.3–1.8), those in the middle of the wealth quintiles (AOR = 1.6, 95% CI 1.1–2.2) and the richer men (AOR = 1.3, 95% CI 1.1–1.8) had higher chances of cigarette smoking than the richest. More so, living in rural area was associated with cigarette smoking as opposed to living in urban localities.

**Table 2:** Adjusted odds ratios (AOR) for cigarette smoking among Ghanaian men in 2003 and 2008 demographic and health surveys

**Indicator**	**Multivariate**	**Multivariate**	**Multivariate model**	Significance of interaction term*
	**2003**	**2008**	**Total sample**	
	AOR* (95% CI)	AOR* (95% CI)	AOR* (95% CI)	
**Age (in years)**				P = 0.001
15–24	1.0	1.0	1.0	
25–34	6.7 (4.2–10.7)	4.4 (2.7–7.3)	5.6 (4.0–7.9)	
35–59	13.2 (8.0–20.7)	8.6 (5.4–13.8)	10.8 (7.8–14.9)	
**Education**				P = 0.026
Higher education	1.0	1.0	1.0	
Secondary education	1.4 (0.7–2.7)	1.6 (0.7–2.8)	1.6 (1.1–2.5)	
Primary education	0.9 (0.5–1.7)	0.9 (0.5–1.6)	0.9 (0.6–1.5)	
No education	2.2 (1.2–4.2)	1.6 (0.8–3.2)	2.2 (1.4–3.4)	
**Occupation**				p = 0.099
Managerial/professional	1.0	1.0	1.0	
Clerical and services	3.0 (1.2–7.4)	3.1 (1.1–8.7)	3.1 (1.6–6.1)	
Manual	5.2 (2.4–11.6)	2.1 (0.8–5.5)	3.7 (2.0–6.8)	
Agriculture	3.4 (1.4–7.9)	4.0 (1.6–10.3)	4.1 (2.2–7.6)	
Not working	5.8 (2.6–13.0)	2.7 (1.1–7.0)	4.2 (2.3–7.6)	
**Religion**				P = 0.228
Christianity	1.0	1.0	1.0	
Traditional religion	2.1 (1.5–3.0)	1.9 (1.4–2.6)	2.6 (2.1–3.3)	
Muslim	2.6 (1.9–3.5)	2.9 (2.0–4.3)	2.9 (2.0–4.2)	
Other religions	**	2.7 (1.8–4.1)	2.0 (1.6–2.5)	
**Labour market position**				P = 0.898
Not in labour force	1.0	1.0	1.0	
In labour force	3.0 (1.7–5.2)	2.1 (1.1–4.2)	2.6 (1.7–4.0)	
**Wealth index**				P = 0.041
Richest	1.0	1.0	1.0	
Richer	1.0 (0.7–1.5)	1.9 (1.2–3.0)	1.3 (1.1–1.8)	
Middle	1.1 (0.7–1.6)	2.8 (1.6–4.6)	1.6 (1.1–2.2)	
Poorer	1.6 (1.0–2.5)	2.5 (1.4–4.5)	1.9 (1.3–2.8)	
Poorest	2.3 (1.4–3.8)	4.8 (2.6–8.6)	3.1 (2.1–4.5)	
**Place of residence**				P = 0.033
Urban	1.0	1.0	1.0	
Rural	0.7 (0.5–1.0)	0.8 (0.6–1.2)	0.8 (0.6–1.0)	

*Interaction between variable and survey year **AOR = 0.00001.

**Figure 1:**
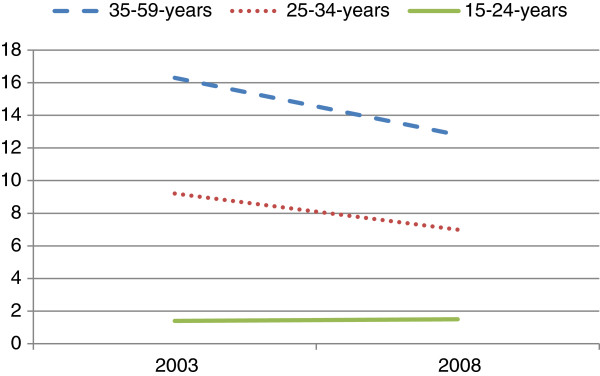
Prevalence of cigarette smoking from 2003 to 2008 among Ghanaian males in relation to age in the demographic and health surveys.

**Figure 2:**
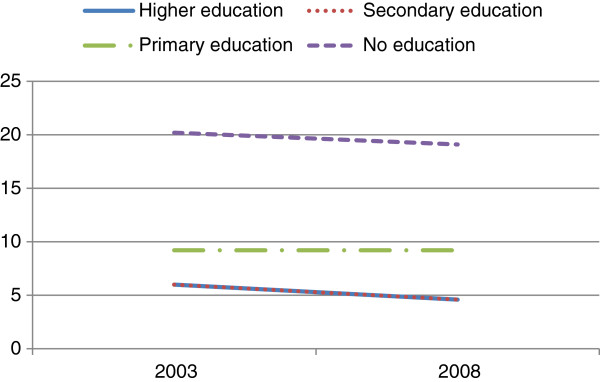
Prevalence of cigarette smoking from 2003 to 2008 among Ghanaian males in relation to educational attainment in the demographic and health surveys.

**Figure 3:**
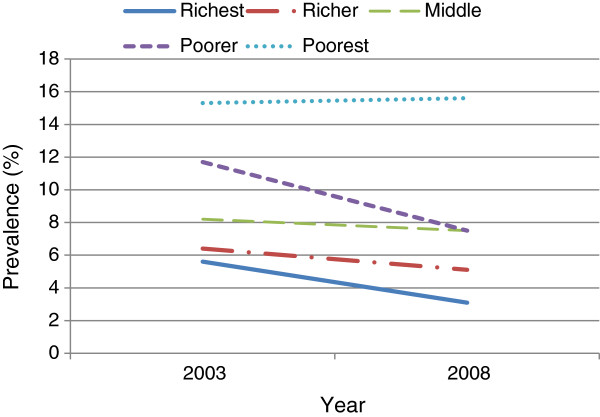
Prevalence of cigarette smoking from 2003 to 2008 among Ghanaian males in relation to wealth index in the demographic and health surveys.

**Figure 4:**
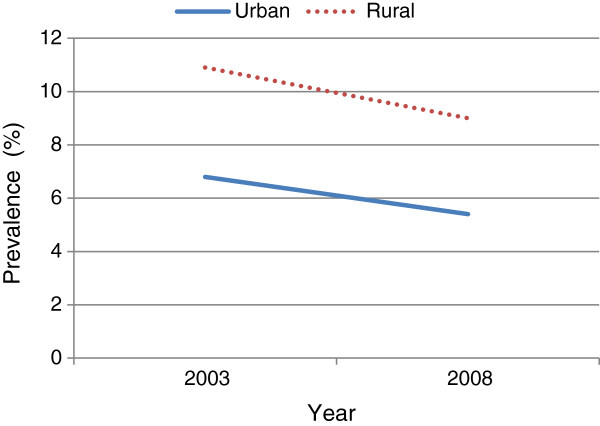
Prevalence of cigarette smoking from 2003 to 2008 among Ghanaian males in relation to place of residence in the demographic and health surveys.

Statistically significant interactions were found between the study year and age, education, wealth and place of residence (Table 2). When the changes in cigarette smoking by these socioeconomic indicators were examined graphically, the following were found:

Over the study periods, it was observed that cigarette smoking decreased significantly among the 25–34 year old and 35–59 year old men, while no decrease was observed among the youngest group (15–24 year group) (Figure 1). Stratified analysis revealed that cigarette smoking decreased among all education groups over time except those who had primary school educational attainment (Figure 2). By wealth, the prevalence of cigarette smoking decreased among all groups in 2008 except the poorest. Additionally, although cigarette smoking among rural dwellers remained high in 2008, the decrease in the phenomenon was more marked in those settings than in urban areas (Figure 3). In the like manner, it was observed that over the period, cigarette smoking decreased slightly more among those with higher and secondary school educational attainments than among those with primary school education and those without formal education (Figure 4).

## Discussion

Cigarette smoking among Ghanaian men is relatively low and seems to be declining. This study has revealed clear socioeconomic differences in cigarette smoking by occupation, labour market position and wealth to the disadvantage of those at the lower end. Similarly, having lower educational attainment increased the likelihood of cigarette smoking. Age, place of residence and religious practice were also related with cigarette smoking among Ghanaian men. Cigarette smoking from 2003 to 2008 revealed that changes in the behaviour over the 5 year period varied by educational attainment, wealth, place of residence and age.

Compared to most Sub-Saharan African (SSA) countries, cigarette smoking is relatively low among Ghanaian men [17]. The prevalence is lower than those reported based on the demographic and health surveys in most Southern African nations including Mozambique (14.1%), Lesotho (15.6%), Zambia (15.6%), and Namibia (17.5%), as well as in Eastern African countries such as Rwanda (14.2%), Uganda (18.7%), Tanzania (21.0%), and Kenya (22.9%). The cigarette smoking prevalence in Ghana is however higher than those reported in Ethiopia (8.3%) and Nigeria (8.0%). The reason why the use of cigarettes is relatively low in Ghana is unknown. Cigarette initiation is mostly socially driven. It is therefore possible that some socio-cultural mechanisms operate in protecting the Ghanaian society from the cigarette epidemic.

Nonetheless, age differences in cigarette smoking have been found in this study. Other studies have reported similar age differences [11,17]. For example, in 16 SSA countries, tobacco use was reported to increase with age till it reaches a peak of about age 40 before declining [17]. Age differences in cigarette smoking are manifestations of cohort effect of the uptake of the behaviour. In Ghana, historically, cigarette smoking is said to have been introduced into the country by veterans who learnt the behaviours during their involvement in the World War II (WWII) overseas. It was therefore expected that cigarette smoking will be more prevalent among the post WWII generation. A recent study reported that tobacco companies are enrolling the youth into smoking through aggressive marketing strategies [12]. If these strategies of the tobacco companies continue, then the expected cohort effect of cigarette smoking among the post WWII generation is likely to change.

Occupation is related to a person’s standing in society [18] and to both income and education. It determines material living standards, reputation in society, control and autonomy, level of stress and social networks. Occupation therefore affects health behaviours and health [19,20]. In this study those with lower occupational grades were found to be more likely to smoke cigarettes than those with professional and managerial occupational grades. This finding is largely consistent with most previous studies in both developed [21] and developing countries [17]. Similarly, we found that the higher a man’s educational attainment, the less likely that he would use cigarette. This relationship between education and cigarette smoking is also consistent with most previous studies in both developed [21] and developing countries [17]. The mechanisms through which education and occupation affect health behaviours are varied and interrelated. Education equips the individual with knowledge and skills to make informed and better health behaviour choices which positively affect health in the long run. Also, education equips one to cope with stress that may arise from work and daily living [19,20]. Moreover, education is a proxy measure of the material, intellectual, and other resources and to some extent determines the place of residence [19]. Education predicts occupation and income in the future [22]. Therefore these same mechanisms may explain the relationship between cigarette smoking and education, occupation, labour market position as well as place of residence among Ghanaian men.

A number of studies have found differences in tobacco use [23] as well as other health behaviours by religious affiliations [24]. Religious affiliation constitutes social network where not only social support exists but also behaviour is shared. Consequently, belonging to a religious sect that promotes health enhancing behaviours such as no smoking and non-excessive alcohol use motivates pursuing such lifestyles.

Wealth, a measure of affluence in the study population, showed the most remarkable gradient in cigarette smoking. The richer a man was, the more likely that he will not smoke cigarette. The discourse on why people with low income or less wealth are more likely to use tobacco product has not been resolved. One school of thought is that smoking is adopted by those in the lower socioeconomic groups as a way of coping with the stress, shame and humiliation that come with such status [25]. The above hypothesis could explain in part why in a poor country like Ghana the poor were rather more likely to spend their meagre sum of money on cigarette compared to the rich.

Place of residence has been shown to predict smoking [17,26,27], although some review studies have not found consistency in this prediction, for example [23]. Place of residence relates to occupation, education, income and one’s overall status in society. Therefore the relationship between place of residence and cigarette smoking could also be explained by the pathway linking education and occupation discussed above. In addition, disparities in access to health information could account for the differences in smoking by place of residence.

Analysis of the changes indicated that although cigarettes smoking decreased in 2008, the decrease varies by age, education, wealth index and place of residence. With respect to all these indicators, the changes tend to favour those at the favourable end. Previous studies have explained changes in smoking trends over time as consistent with the conceptualisation of how the smoking epidemic diffuses over time [13-16]. A number of observations can be made about the smoking epidemic with regards to the present findings. If indeed the smoking epidemic is in its initial stage in Ghana, then it is possible that the pattern of the epidemic is not following the same course as observed in developed countries where it begins with the upper social class, diffuses to the entire population before accumulating in the lower social class [15]. On the other hand, if we assume that the epidemic is in its final stage, then, again, it seems that it has not gone through the peak stage which is a typical nature of the epidemic. Tobacco use in Ghana and perhaps many Sub-Saharan African countries is relatively young. In Ghana for example, the behaviour is believed to have been introduced into the country after the World War II. In this respect, it is possible that the pattern is not following the classical model observed in developed countries because at the start of the menace, the health damaging effects were already known and therefore those in the higher socioeconomic groups (the innovators) in developing countries did not begin. More studies from developing countries, particularly Sub-Saharan Africa, are needed to increase our understanding on the pattern of the smoking epidemic in the region. Such studies will be important for curbing the menace, especially in those countries that are experiencing rising prevalence of tobacco use.

## Conclusion

Cigarette smoking among Ghanaian men is relatively low and seems to be decreasing over the years. However, socioeconomic differences exist in cigarette smoking mainly to the disadvantage of those in the lower socioeconomic groups. Between 2003 and 2008, cigarette smoking seems to have decreased in the male population but not among all groups by age and socioeconomic status assessed by education, wealth and place of residence. Tobacco control interventions should be tailored to reduce the menace in all SES groups, particularly those with no formal education and the poorest in the society.

## Competing interests

The authors declare that they have no competing of interests.

## Authors’ contributions

DD conceived the study and drafted the first version of the manuscript. DD, EKMD and AKK revised the manuscript for important intellectual content and gave consent for the version to be published. All authors have read and approved the final manuscript.
